# Clinical implications of adopting Monte Carlo treatment planning for CyberKnife

**DOI:** 10.1120/jacmp.v11i1.3142

**Published:** 2010-01-29

**Authors:** Subhash C. Sharma, Joseph T. Ott, Jamone B. Williams, Danny Dickow

**Affiliations:** ^1^ Parkview Comprehensive Cancer Center Fort Wayne IN USA

**Keywords:** CyberKnife, Monte Carlo, treatment planning

## Abstract

It is documented that well‐modeled Monte Carlo dose calculation algorithms are more accurate than traditional correction‐based algorithms or convolution algorithms at predicting dose distributions delivered to heterogeneous volumes. This increased accuracy has clinical implications for CyberKnife, particularly when comparing dose distributions between the ray‐tracing and Monte Carlo algorithms. Differences between ray‐tracing and Monte Carlo calculations are exacerbated for highly heterogeneous volumes and small field sizes. In this study, the anthropomorphic thorax phantom from the Radiological Physics Center was used to validate the accuracy of the CyberKnife Monte Carlo dose calculation algorithm. Retrospective comparisons of dose distributions calculated by ray‐tracing and Monte Carlo were made for a selection of CyberKnife treatment plans; comparisons were based on target coverage and conformality. For highly heterogeneous cases, such as those involving the lungs, the ray‐tracing algorithm consistently overestimated the target dose and coverage. In our sample of lung treatment plans, the average target coverage for ray‐tracing calculations was 97.7%, while for Monte Carlo, the average coverage dropped to 69.2%. In each plan comparison, the same beam orientations and monitor units were used for both calculations. Significant changes in conformality were also observed. Isodose prescription lines and subsequent target coverage selected for treatment plans calculated with the ray‐tracing algorithm may be different from comparable treatment plans calculated with Monte Carlo, and as such, may have clinical implications for dose prescriptions.

PACS number: 87.53.Wz

## I. INTRODUCTION

The CyberKnife robotic radiosurgery system (Accuray, Sunnyvale, CA) is routinely used in the treatment of early stage and/or inoperable lung cancer, in addition to intracranial tumors, which can be treated by many radiosurgical modalities. Radiosurgical accuracy is achieved through the use of frameless real‐time image guidance and dynamic tumor tracking throughout the entire respiratory cycle.^(1)^ Field sizes are set by interchangeable secondary circular collimators ranging in diameter from 5.0 mm to 60 mm. Lung cancer treatments generally consist of three to four fractions, delivering a total dose of 4500 to 6000 cGy.

Despite the highly accurate targeting capabilities of CyberKnife, until recently the only dose calculation algorithm available for CyberKnife treatment planning was a simple ray‐tracing pathlength correction algorithm. In the ray‐tracing algorithm, off‐center ratios (OCR), tissue‐phantom ratios (TPR), and collimator output factors (OF), measured under reference conditions, are corrected for patient geometry. Tissue heterogeneities are accounted for through a central axis effective depth calculation $\left(d_{\text{eff}}\right)$; there are no corrections for changes in electron transport or lateral scatter disequilibrium that may develop in the presence of low‐density heterogeneities. The following equation represents the calculation of dose output (cGy/MU) for the ray‐tracing algorithm currently used in CyberKnife treatment planning:^(2)^

(1) $${\;}^{D}{/}_{MU}=\text{OCR}(\text{col}l,R_{800},d_{\text{eff}})\cdot {\left(\frac{800}{\text{SAD}}\right)}^{2}\cdot \text{TPR}(FS,d_{\text{eff}})\cdot OF(\text{col}l,\text{SAD})$$

The ray‐tracing algorithm is quite sufficient for dose calculations involving homogeneous structures such as the brain; however, more robust heterogeneity corrections are required for accurate dose calculations in highly heterogeneous structures such as the lungs.^(3)^ Tissue heterogeneity effects are exacerbated for small field sizes, which are characteristic of CyberKnife and other stereotactic radiosurgery systems.^(^ ^4^ ^,^ ^5^ ^,^ ^6^ ^)^ To address these issues, a Monte Carlo dose calculation algorithm is now commercially available for CyberKnife treatment planning. This algorithm utilizes a dual source model with parameters derived from clinically measured central axis percent depth dose curves, beam profiles, and collimator output factors.^(7)^ Tissue heterogeneities are inherently included in the Monte Carlo algorithm through the calculation of particle interaction probabilities and subsequent dose deposition.

The increased accuracy of Monte Carlo dose calculation may have clinical implications for CyberKnife dose prescriptions. Protocols for CyberKnife treatment planning, which use dose distributions calculated with the ray‐tracing algorithm, may need to be reevaluated for Monte Carlo calculations. In this study, we aim to quantify the differences expected between ray‐tracing and Monte Carlo calculations for CyberKnife treatment planning.

## II. MATERIALS AND METHODS

The Radiological Physics Center (RPC) anthropomorphic thorax phantom was used to validate the accuracy of the CyberKnife Monte Carlo dose calculation algorithm (Multiplan 2.1.0). The RPC thorax phantom is designed to simulate the treatment of a solitary left lung mass.^(8)^ Dosimetry inserts allow comparisons between planned and delivered dose distributions; thermoluminescent dosimetry capsules are used for absolute dose comparisons and radiochromic film is used for relative dosimetry.

To evaluate the clinical implications of adopting Monte Carlo treatment planning for CyberKnife, a retrospective comparison was made between dose distributions calculated by the ray‐tracing and Monte Carlo algorithms. A sample of 18 lung treatment plans, treated with CyberKnife at Parkview Comprehensive Cancer Center, was selected for this comparison. The lung plans were designated as either peripheral or central according to the guidelines from the Lung Cancer Stereotactic Radiotherapy vs. Surgery (STARS) Trial. Targets are considered peripheral if they reside greater than two centimeters from mediastinal, pulmonary, and vertebral structures; all other targets are considered central. In each case, a clinically acceptable treatment plan was developed using the ray‐tracing algorithm. The beam sets for each ray‐tracing plan were then recalculated using the Monte Carlo algorithm (Gaussian smoothing, 1%). In this fashion, the beam orientations and monitor units for each respective pair of calculations were identical, only the resulting dose distributions changed.

Comparisons between the Monte Carlo and ray‐tracing dose distributions were based on the dose statistics parameters available in Multiplan: prescription dose coverage, conformality index (CI), and new conformality index (nCI). These are some of the same parameters typically used to judge whether or not a plan is clinically acceptable for treatment. Prescription dose coverage is simply defined as the fraction of the tumor volume that receives at least the prescription dose. The CI represents the ratio of the tissue volume receiving at least the prescription dose to the tumor volume receiving at least the prescription dose, while the nCI is the CI multiplied by the ratio of the total tumor volume to the tumor volume covered by the prescription isodose line.

## III. RESULTS

### A. RPC thorax phantom irradiation

**Figure 1:**
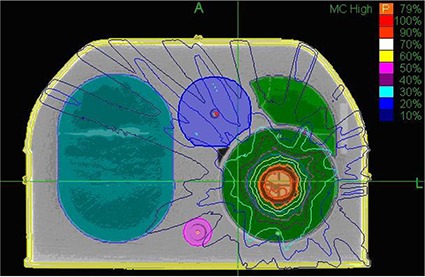
Representative image from the Monte Carlo validation treatment plan, using the RPC thorax phantom. The target (PTV) is located in the left lung; 600 cGy is prescribed to the 79% isodose line.

A clinically acceptable treatment plan, calculated with the Monte Carlo algorithm, was developed for the thorax phantom following the dose prescription and planning guidelines provided by the RPC. A dose of 6 Gy, prescribed to the 79% isodose line, was delivered to the planning target volume (PTV) (Fig. 1). The RPC criteria for PTV coverage are 6 Gy and 5.4 Gy to at least 95% and 99% of the PTV, respectively. In addition, doses delivered to the critical structures (heart, spinal cord, healthy lung) were all well below the tolerance values. For analysis purposes, the PTV structure contains two sets of TLD capsules and radiochromic films oriented in the axial, sagittal, and coronal planes. Results of the TLD and film analysis for the PTV are contained in Table 1. The TLD values represent the ratio of the measured dose to the dose predicted in the Monte Carlo treatment plan. The film values represent the average displacement of the dose gradient on each side of the PTV, in each plane.

**Table 1:** TLD and film results for the Monte Carlo validation plan using the RPC thorax phantom.

*Location*	*RPC vs. Plan*	*Criteria*
PTV TLD 1	1.00	0.92 – 1.02
PTV TLD 2	0.96	0.92 – 1.02
Left/Right Film	0/3 mm	≤5 mm
Post/Ant Film	1/4 mm	≤5 mm
Inf/Sup Film	1/1 mm	≤5 mm

### B. Monte Carlo vs. ray‐tracing

In every lung treatment plan that was analyzed, the ray‐tracing algorithm overestimated the dose and coverage of the target volume. Table 2 contains a summary of the ray‐tracing and Monte Carlo calculations for the lung plans, with σ representing the standard deviation of each parameter. The average target coverage for the ray‐tracing plans was 97.7%±1.7%, while for the Monte Carlo calculations, the average coverage dropped to 69.2%±15.2% for the same isodose prescription line. A significant change in conformality was also observed, which again indicates the inaccuracy of the ray‐tracing algorithm in heterogeneous volumes.

**Table 2:** Summary of coverage and conformality comparisons between the ray‐tracing and Monte Carlo treatment plans. Significant degradation is observed for the Monte Carlo plans.

		*Ray‐Tracing Calculations*			*Monte Carlo Calculations*		
	*Rx Isodose (%)*	*Coverage (%)*	*CI*	*nCI*	*Coverage (%)*	*CI*	*nCI*
Average	84	97.7	1.27	1.30	69.2	1.12	1.72
σ	2	1.7	0.09	0.09	15.2	0.06	0.59
%σ	2.4	1.7	7.0	6.7	22.0	5.4	34.3

To achieve target coverage comparable to that attained for the ray‐tracing plans, the Monte Carlo calculations required a prescription line of 75%±4%, a drop of 9% when compared to the ray‐tracing algorithm (Table 3. A lower isodose prescription line results in an increased CI, indicating a much less conformal plan, with more healthy tissue receiving the prescription dose. The maximum dose within the plan also increases proportionately with a decrease in the prescription isodose line. For example, decreasing the prescription isodose line from 83% to 75% would increase the maximum dose “hot spot” by approximately 770 cGy, for a 6000 cGy prescription.

**Table 3:** Comparison between the average isodose prescription lines required for comparable ray‐tracing and Monte Carlo target coverage, and subsequent conformality indexes for the Monte Carlo plans.

	*Ray‐Tracing Rx Isodose (%)*	*Rescaled Monte Carlo Isodose (%)*	*CI*	*nCI*
Average	84	75	1.47	1.51
σ	2	4	0.22	0.21
%σ	2.4	5.3	15.0	13.9

There is also a shift in the standard deviation of each of the parameters listed in Tables 2 and 3; in general, a much wider range of coverage and nCI was observed for the Monte Carlo calculations. This indicates that, although there was a uniform drop in target coverage and conformality after the Monte Carlo calculations, some plans were affected more significantly than others.

**Figure 2:**
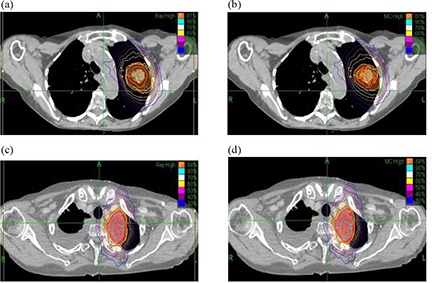
Comparison between ray‐tracing (left) and Monte Carlo (right) treatment plans exhibiting the importance of tumor position within the lung as a predictive factor. In each case, the prescription isodose line appears as the thick orange line: 87% for the peripheral target and 84% for the central target.

Several factors could potentially influence the discrepancy observed between ray‐tracing and Monte Carlo calculations including tumor location and PTV composition. Figure 2 presents an example of two lung treatments where tumor location appears to greatly influence the accuracy of the ray‐tracing calculation. The first case (Figs. 2(a) and (b)) is a peripheral tumor surrounded on all sides by low‐density lung tissue; in fact, a significant percentage of the volume of the PTV consists of lung tissue. The second case (Figs. 2(c) and (d)) is a central lesion located along the mediastinum and extending into the apex of the lung, where fewer beams traverse the low‐density lung tissue. The volumes of the two tumors are approximately the same; however, when comparing the respective ray‐tracing and Monte Carlo calculations, the coverage drops to 41.3% for the peripheral tumor and 90.6% for the central tumor. In each case, there was more than 95% coverage of the PTV according to the ray‐tracing plan.

## IV. DISCUSSION & CONCLUSIONS

**Figure 3:**
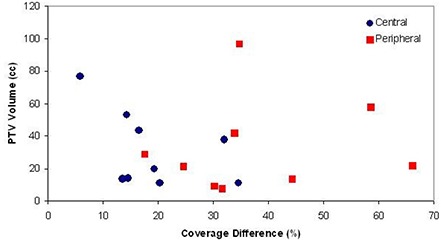
Scatter plot indicating the difference in ray‐tracing and Monte Carlo coverage observed for the sample of 18 lung treatment plans

There can be significant differences between ray‐tracing and Monte Carlo calculations for CyberKnife treatment planning, particularly for treatments involving heterogeneities such as the lungs. This is due to the tendency of the ray‐tracing algorithm to overestimate the dose to the target for heterogeneous volumes. The magnitude of overestimation is influenced by the location of the tumor within the lung and amount of lung tissue contained within the target volume. On average, in our sample of lung treatment plans, the Monte Carlo calculations resulted in a decrease in target coverage of approximately 28%±15%, as compared to the initial ray‐tracing calculations. Considerable spread was observed in the coverage difference between ray‐tracing and Monte Carlo for our sample of lung treatment plans (Fig. 3). In general, the greatest discrepancy was observed for peripheral lung tumors; however, there did not seem to be a well‐defined pattern.

Stereotactic lung radiosurgery protocols that currently specify ray‐tracing as the appropriate dose calculation algorithm may need to evaluate a reduced dose prescription when transitioning to the Monte Carlo algorithm. For example, a 6000 cGy prescription, calculated with the ray‐tracing algorithm, may actually be delivering significantly less dose to large portions of the target. The discrepancy between ray‐tracing and Monte Carlo dose distributions may need to be investigated on a case‐by‐case basis. There does not seem to be a uniform scaling factor that can be used to accurately transform a ray‐tracing dose prescription into an equivalent Monte Carlo dose prescription for all cases.

## ACKNOWLEDGEMENTS

The authors thank Mu Young Lee, Ph.D. and Miroslav Nikolic, Ph.D. of Accuray, Inc.
